# Predicting sex from retinal fundus photographs using automated deep learning

**DOI:** 10.1038/s41598-021-89743-x

**Published:** 2021-05-13

**Authors:** Edward Korot, Nikolas Pontikos, Xiaoxuan Liu, Siegfried K. Wagner, Livia Faes, Josef Huemer, Konstantinos Balaskas, Alastair K. Denniston, Anthony Khawaja, Pearse A. Keane

**Affiliations:** 1 NIHR Biomedical Research Center at Moorfields Eye Hospital NHS Foundation Trust and UCL Institute of Ophthalmology, London, UK; 2 Department of Ophthalmology, University Hospitals Birmingham NHS Foundation Trust, Birmingham, UK; 3 Academic Unit of Ophthalmology, Institute of Inflammation & Ageing, College of Medical and Dental Sciences, University of Birmingham, Birmingham, UK; 4 Eye Clinic, Cantonal Hospital of Lucerne, Lucerne, Switzerland; 5 Vienna Institute for Research in Ocular Surgery, A Karl Landsteiner Institute, Hanusch Hospital, Vienna, Austria; 6 Health Data Research UK, London, UK

**Keywords:** Translational research, Computer science

## Abstract

Deep learning may transform health care, but model development has largely been dependent on availability of advanced technical expertise. Herein we present the development of a deep learning model by clinicians without coding, which predicts reported sex from retinal fundus photographs. A model was trained on 84,743 retinal fundus photos from the UK Biobank dataset. External validation was performed on 252 fundus photos from a tertiary ophthalmic referral center. For internal validation, the area under the receiver operating characteristic curve (AUROC) of the code free deep learning (CFDL) model was 0.93. Sensitivity, specificity, positive predictive value (PPV) and accuracy (ACC) were 88.8%, 83.6%, 87.3% and 86.5%, and for external validation were 83.9%, 72.2%, 78.2% and 78.6% respectively. Clinicians are currently unaware of distinct retinal feature variations between males and females, highlighting the importance of model explainability for this task. The model performed significantly worse when foveal pathology was present in the external validation dataset, ACC: 69.4%, compared to 85.4% in healthy eyes, suggesting the fovea is a salient region for model performance OR (95% CI): 0.36 (0.19, 0.70) p = 0.0022. Automated machine learning (AutoML) may enable clinician-driven automated discovery of novel insights and disease biomarkers.

## Introduction

The retina is the only tissue in the body where neural and vascular tissue can be visualized simultaneously in a non-invasive manner. Ophthalmologists have been doing so since the ophthalmoscope was introduced into clinical practice in the mid 1800s^1^. It has also been increasingly recognized that retinal biomarkers may map effectively to systemic indices of healthy ageing and disease^2–6^. Examples of these oculomics-based findings include vascular tortuosity and arteriolar narrowing for cardiovascular disease, and retinal cell layer changes for neurological disorders^7–11^.

Relationships between retinal morphology and systemic health have traditionally been evaluated using statistical modelling, such as multivariable regression. However, such techniques may have limited incremental value when leveraged on very large datasets and for complex data^12,13^. As data availability has increased, and mathematical models have improved, the success of deep learning in ophthalmic disease classification in the research setting has been striking^14–18^. Deep neural networks, which process input images by applying mathematical operations to connected nonlinear units in multiple layers, largely avoid manual feature engineering, and are able to derive previously hidden patterns in large volumes of data^19^.

The discovery of quantitative relationships between retinal appearance and systemic pathophysiology readily aligns with pre-established conceptions of microvascular and degenerative tissue-level insults^20^. However, deep learning has shown that these algorithms demonstrate capability in tasks which were not previously thought possible^21^. Harnessing this power, new insights into relationships between retinal structure and systemic pathophysiology could expand existing knowledge of disease mechanisms. A study by Poplin et al. demonstrated a deep-learning learning algorithm which could accurately predict cardiovascular risk factors from fundus photos^22^; More surprising to ophthalmologists was the successful prediction of demographic information such as age and gender, the latter with an area under the curve (AUC) of 0.97. Here, the physiologic cause and effect relationships are not readily apparent to domain experts^21^. Predicting gender from fundus photos, previously inconceivable to those who spent their careers looking at retinas, also withstood external validation on an independent dataset of patients with different baseline demographics^23^. Although not likely to be clinically useful, this finding hints at the future potential of deep learning for the discovery of novel associations through unbiased modelling of high-dimensional data.

We previously reported on the ability of physicians to create automated machine learning (AutoML) models for medical image analysis^24^. Since that proof of concept, AutoML platforms have advanced significantly, with multiple employing code free deep learning (CFDL). Herein, we demonstrate AutoML as a tool for automated discovery of novel insights by performing sex classification from retinal fundus photos, and comparing its performance to the bespoke deep learning model by Poplin et al^22^.

## Results

### CFDL model results

**Figure 1:**
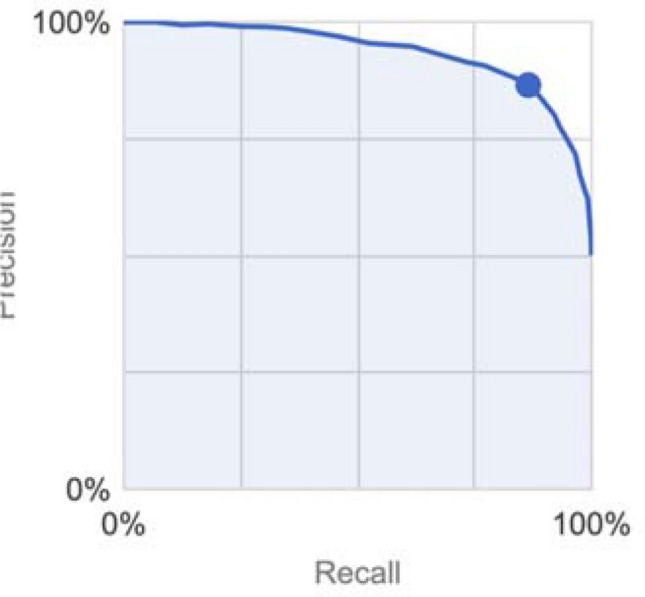
Precision-recall curve.

The CFDL model had an AUROC and AUPRC of 0.93 and 0.94 respectively (Table 1). Overall sensitivity (recall), specificity, PPV (precision), and ACC were 88.8%, 83.6%, 87.3%, and 86.5% respectively (Fig. 1). Genetic sex was discordant from reported sex in one validation set image, and this image was incorrectly predicted by the model; that is the model predicted sex consistent with genetic sex in this case (Table S1). To evaluate reproducibility and address varying performance of deep learning algorithms involving random seed initiation, we retrained the model to identical specifications, and found similar performance with an AUC of 0.93.

**Table 1:** Comparison of reported fundus photo sex prediction algorithms.

Model	AUROC	Dataset source	Training dataset images	Mean training set age	Mean test set age
CFDL	0.93	UK Biobank	173,819	56.8	55.7
Poplin et al	0.97	UK Biobank + EyePACS	1,779,020	54.1	56.6
Yamashita et al	0.78	Kagoshima University Hospital	111*	25.8*	25.8*

*Cross-validation.

### External validation

External validation was performed on the Moorfields dataset. This dataset differed from the UK Biobank development set with respect to both fundus camera used, and in sourcing from a pathology-rich population at a tertiary ophthalmic referral center. The resulting sensitivity, specificity, PPV and ACC were 83.9%, 72.2%, 78.2%, and 78.6% respectively.

### Presence of foveal pathology

**Figure 2:**
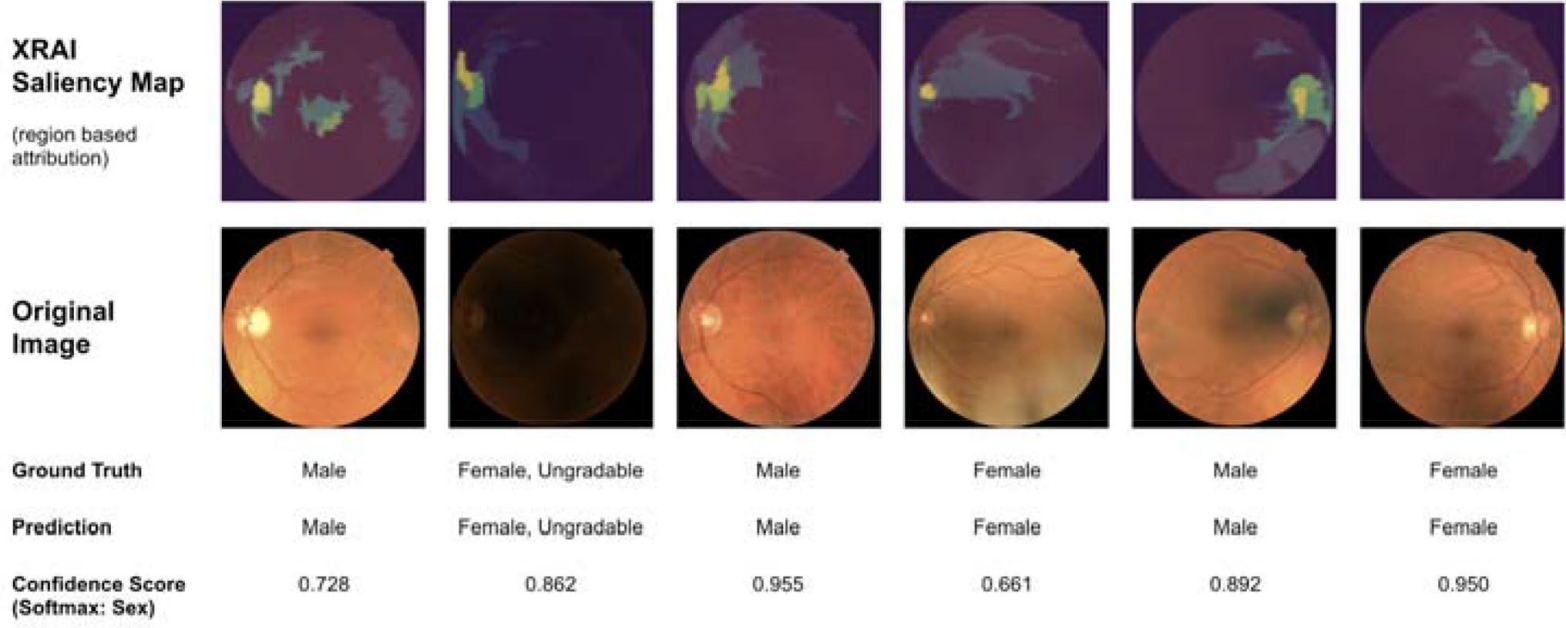
Region based saliency maps for model prediction: colors represent regions in order of decreasing performance: Yellow, Green, Blue. Images sourced at random from validation set, with the addition of an ungradable image.

To evaluate the influence of foveal pathology on the performance of the CFDL model, we subgrouped the Moorfields external validation dataset into fundus photos with (n = 108) and without (n = 144) foveal pathology (Table 2). The model classified sex correctly in 85.4% of patients without foveal pathology, a population more similar to the largely health UK Biobank population, compared to 69.4% in patients with foveal pathology. Logistic regression showed that presence of foveal pathology was a significant factor in model performance OR (95% CI): 0.36 (0.19, 0.70) p = 0.0022. Sex was not associated with presence of foveal pathology (p = 0.94). This suggests that the fovea may be a salient region of fundus photographs for the neural network’s sex classification performance. Region attribution saliency maps suggest the optic nerve and vascular arcades as additional important input regions for the model’s prediction (Fig. 2).

**Table 2:** Model performance on external validation dataset subgrouped by presence of foveal pathology.

	Percent correct prediction	Odds ratio (95% CI) for correct prediction	P-value
**Foveal pathology**			
None	85.4%	Ref	
Present	69.4%	0.36 (0.19, 0.70)	0.0022
Age (years)		1.00 (0.99, 1.02)	0.66

### Ungradable UK biobank images

Consensus ungradable images (n = 714), which were formerly removed from the UK Biobank validation dataset were separately processed by the model as an experimental adjunct batch prediction. The resulting sensitivity, specificity, PPV and accuracy were 82.6%, 71.2%, 75.2%, and 77.0% respectively.

## Discussion

Our results demonstrate robust overall performance of the CFDL model for predicting sex from retinal fundus photos. The AUROC of 0.93 from this framework, which does not require coding expertise, suggests significant capability of the CFDL platform for this task. Our code-free model’s performance is comparable with the Poplin et al. model AUROC of 0.97, which was designed and tuned by machine learning experts (Table 1). Our model was trained on a similar (UK Biobank), albeit significantly smaller dataset, as it did not include the additional 1.5 + million EyePACS fundus photos which Poplin et al. also utilized for training.

To our knowledge, two other studies have attempted to perform this image classification task. Yamashita et al. performed logistic regression on several features that were identified to be associated with sex. These features included papillomacular angle, retinal vessel angles and retinal artery trajectory^25^. They achieved an AUROC of 0.78, which further underscores the limitations of a classical machine learning approach, utilizing human-identified features for such novel tasks. Deep learning, even utilizing our CFDL approach, seems to outperform manual feature engineering significantly. Various studies have shown retinal morphology differences between the sexes, including retinal and choroidal thickness^26–28^. Others have demonstrated variation of ocular blood flow and have suggested the effect of sex hormones, but thus far, consensus is lacking^29,30^. The coder-engineered deep learning model developed by Dieck et al. for this task, which also included an image preprocessing step, demonstrated an accuracy of 82.9%, which was lower than our automated code-free approach^31^. The retinal features apparent to domain experts for this task may go unanswered, as the power of deep learning in integrating population-level patterns from billions of pixel-level variations is impossible for humans to match.

Performance of our model was slightly worse with external validation on the Moorfields dataset, which is typical when deep learning models are evaluated with datasets dissimilar from their training data distribution^32,33^. Specifically, the Moorfields dataset was obtained from a tertiary referral center, and 42.9% of the fundus photos contained foveal pathology. In eyes without foveal pathology, the external validation accuracy was within 1.5% of the Biobank validation set. The worse performance in pathologic eyes suggests the significance of the fovea for sex prediction, and was similarly demonstrated in the attention maps of Poplin et al. The region-based saliency maps we generated suggest that the optic nerve and vascular arcades are also regions of importance (Fig. 2). In the study by Poplin et al., when subgrouped for diabetic retinopathy (DR) presence, their model similarly trended towards worse performance for pathologic images compared with healthy controls. Furthermore, the ophthalmologists in that study “repeatedly reported highlighting of the macula for the gender predictions” when interpreting the attention maps^22^. These findings highlight the importance of considering machine learning performance only in context of the specific training and evaluation datasets utilized. This is especially critical for our task, when the salient features of an input image are unclear to domain experts.

Ungradable images from the UK Biobank validation dataset were labeled as such by retina specialists to the guidelines of lacking adequate visibility of the macula, optic nerve, and vascular arcades (Table S2). However, those images demonstrated only a slight reduction in model performance. Furthermore, the model shows similar salient regions in ungradable input images as in gradables (Fig. 2). This suggests that the model is sensitive to signal in poor quality images from subtle pixel-level luminance variations, which are likely indifferentiable to humans. This finding underscores the promising ability of deep neural networks to utilize salient features in medical imaging which may remain hidden to human experts.

Through characterization of high-dimensional data, our findings suggest that deep learning will be a useful tool for the exploration of novel disease and biomarker associations. Clinician driven research, particularly through the use of AutoML, has the potential to move this field forward. Crucially, AutoML as a platform does not fully automate the process of machine learning. Data preparation remains an essential manual step. As demonstrated by population differences in our external validation dataset, tasks such as equitable and representative acquisition, cleaning, and subgrouping of datasets remain important factors for the production of useful models. Clinicians are uniquely positioned to understand both the complexities of the clinical data, and the use-cases for the design of clinically relevant production algorithms.

While our deep learning model was specifically designed for the task of sex prediction, we emphasize that this task has no inherent clinical utility. Instead, we aimed to demonstrate that AutoML could classify these images independent of salient retinal features being known to domain experts, that is, retina specialists cannot readily perform this task. We intended to show that our framework’s performance may be comparable to state of the art algorithms designed for the same task by coders. This portends for the capacity of AutoML, utilized by clinician use-case experts, to design models for tasks where specific retinal features have not been categorized. Examples of such use-cases include cardiovascular and neurological disease characterization from retinal photos.

### Limitations

Our study had several limitations. The design of the CFDL model was inherently opaque due to the framework’s automated nature with respect to model architecture and hyperparameters. While this opacity is not unique to CFDL, there is potential to further reduce ML explainability due to lack of insight of model architectures and parameters employed. Although we compared our performance to other models via AUROC, we were unable to compare performance using clinically relevant metrics such as sensitivity and specificity, as these were not provided by the authors of the other studies^34^. The UK Biobank dataset, composed of a generally healthy Caucasian population, was not fully representative of the general UK population, and demonstrates potential for algorithmic bias^35–37^. Although we attempted to address this with an external validation population with a higher prevalence of pathology, our patient level data was limited and did not include additional demographic information. Since both datasets were from UK populations and de-identified, there is the potential of overlap at the patient level.

Through our investigations of predicting other novel systemic signals from fundus photos, we noted several inherent limitations of the CFDL platform. Utilizing buckets of varying range, which were necessary due to lack of support for continuous variable prediction, we were unable to successfully predict age. Experiments to predict smoking status resulted in models with significantly lower AUC (0.64) as compared with Poplin et al. (0.71)^22^. We have engaged the platform development team, and aim to repeat our experiments as new platform features are released.

## Conclusion

We demonstrate clinician-driven design of a deep learning sex classification model from retinal fundus photographs, and comparable performance to the same task in a landmark study. In contrast to the latter model designed by expert engineers, our model was created by clinicians without coding. Our external validation on a population with high levels of foveal pathology suggests that the foveal region is important for this task. This demonstrates AutoML as a tool for novel insight discovery for medical imaging by its clinician end users. Although ophthalmologists may continue to ponder what these deep learning models are “looking at”, our study demonstrates the robust potential of CFDL to characterize images independent of experts’ knowledge of contributing features, and its ability to democratize access to deep learning.

## Methods

### Participants and data

This work utilized two datasets. For deep learning model development, we used the UK Biobank dataset, which is an observational study in the United Kingdom that began in 2006 and has recruited over 500,000 participants—85,262 of which received eye imaging^38^. Eye imaging was obtained at 6 centers in the UK and comprises over 10 terabytes of data^39^. Participants volunteered to provide data including other medical imaging, laboratory results, and detailed subjective questionnaires. Consent was provided by each participant, and the study was approved by the North West Multi-Centre Research Ethics Committee. Detailed protocols may be located at www.ukbiobank.ac.uk. Retinal imaging was obtained with a Topcon OCT-1000 MKII. Each capture consisted of optical coherence tomography and a paired 45-degree retinal fundus photograph. The UK Biobank fundus photo dataset of 175,825 images was split chronologically to train, tuning, and validation sets (Table 3). The train/tuning and validation sets contained 53.6% and 56.0% reported women respectively. For temporal validation, chronologic splits were performed to simulate model performance on the validation set in a manner which would align with a prospective trial^40^; that is a model trained on the first chronological period of data (training dataset), and then evaluated on the subsequent chronological period of data (validation dataset). Participant level splits were preserved throughout—each participant’s left, right, and repeat photos were never split between image subsets.

**Table 3:** Dataset characteristics of UK biobank and moorfields external validation sets.

	UK Biobank development (train + tuning)	UK Biobank validation	Moorfields external validation
Patients	84,743	728*	252
Images	173,819	1287*	252
Mean age	56.8	55.7	64.0
St. Dev. age	8.0	8.1	17.7
Gender (% female)	53.6%	56.0%	54.2%

*Numbers reported are post-removal of ungradable images.

For external validation, we utilized an anonymized clinical dataset convenience sampled from Moorfields Eye Hospital of 400 adult patients. These patients received 45-degree fundus photography with Topcon OCT-2000 in December 2019. In order to obtain a representative dataset of all patients presenting from that time period, no other filters were applied. Both datasets consisted of 50% left and 50% right eyes. Average age in the external validation dataset was 64.0 as compared with the UK Biobank validation dataset average of 55.7 (Table 3). Input of the external validation dataset into Cloud AutoML was through the Moorfields Eye Hospital Research Informatics Strategy Data Platform, a secure cloud-based infrastructure facilitating storage and processing of anonymized clinical data. Project-specific approval from local information governance was granted following submission of a Cloud AutoML data privacy impact assessment and separate dataset-specific treatment standard operating procedure. Research and development approval by the Institutional Review Board at Moorfields was obtained (ROAD17/031). Local and national research opt-outs were queried, and the corresponding patients were excluded. All methods were performed in accordance with the relevant guidelines and regulations.

Participants in the UK Biobank study have provided written informed consent. The external validation set is part of a retrospective, non-interventional study on de-identified data. National and local opt-out guidance was followed for anonymized datasets, Moorfields information governance waived the requirement for informed consent accordingly.

### Data processing and labeling

The gender variable, as described by the UK Biobank, was acquired from the central registry at recruitment, but in some cases was updated by the participant. Therefore, this field may contain a mixture of NHS recorded gender and self-reported gender. Genetic sex in the UK Biobank was determined by genotyping performed at Affymetrix3 Inc. with quality control of the data at the Wellcome Trust Centre for Human Genetics.

**Figure 3:**
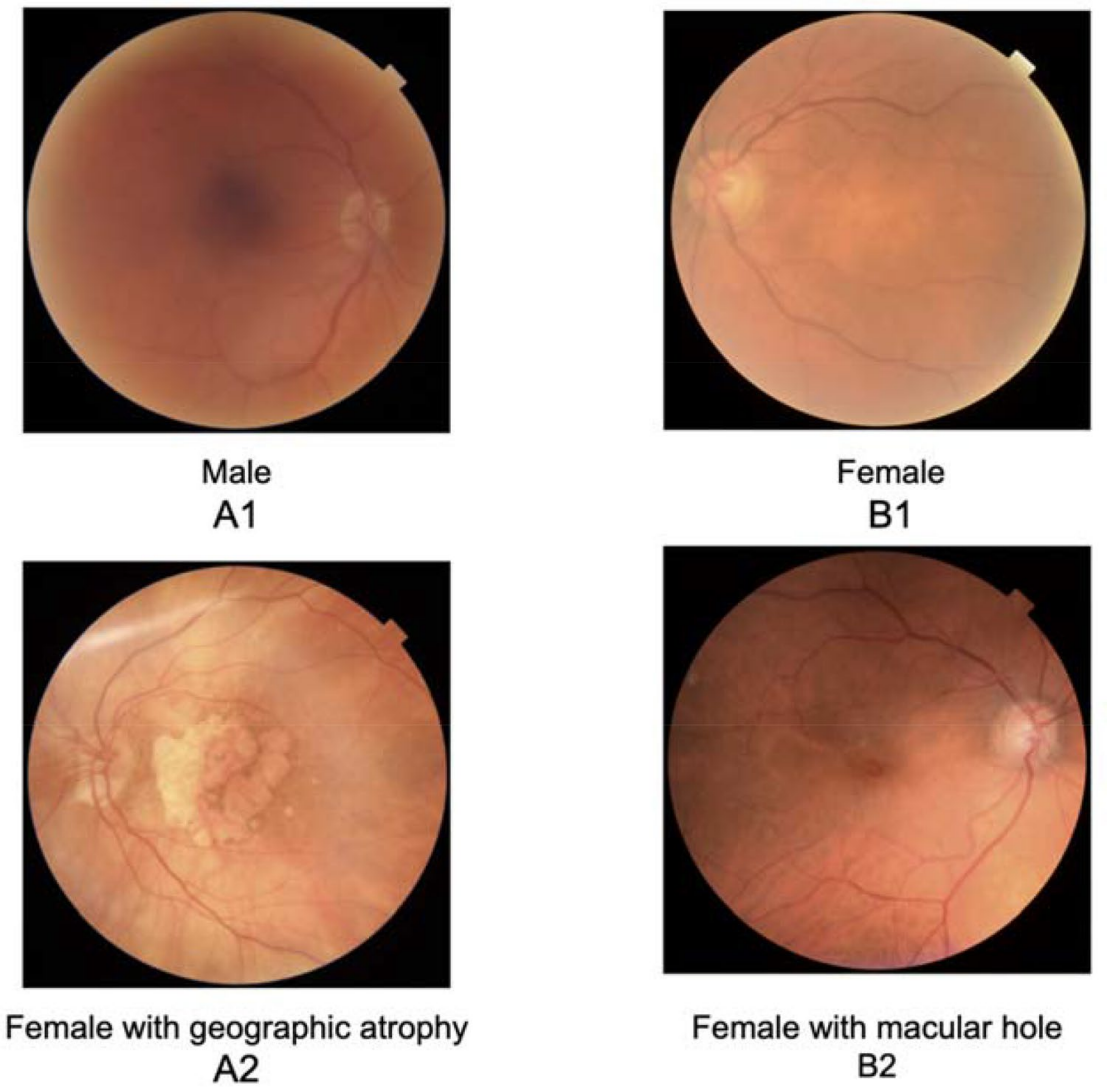
Representative Fundus Photos. Correct (**A**) and incorrect (**B**) cases without (1) and with (2) foveal pathology.

Ungradable images were removed from both validation datasets. De-identified images were assessed for gradability by two retina specialists masked to patient demographics (E.K., H.K.). Gradability was defined as a field of view ensuring adequate visibility of the vascular arcades, macula, and optic nerve, and sufficient image quality to exclude microaneurysm sized features. Any disagreements were resolved via in-person discussion. In cases where agreements could still not be resolved (n = 122), a gradability algorithm, described in a model card (Table S2), was used to adjudicate disagreements, with 70 of the disagreed images being classified as ungradable^41^. After ungradables were removed, 252 Moorfields images remained for external validation. Ungradable rate was 35.7% and 32.2% in the UK Biobank validation and Moorfields datasets respectively. Moorfields images were also graded for presence of foveal pathology. This was defined as any retinal lesion which extended into the central one disc diameter around the fovea. Examples of foveal lesions included but were not limited to macular holes, microaneurysms, RPE tears, and pigment atrophy (Fig. 3).

### Model training

Our deep learning model was trained using code-free deep learning (CFDL) with the Google Cloud AutoML platform. As described and demonstrated in a supplemental video of our prior work^24^, the platform provides a graphical user interface (GUI) for data upload, labeling, and model training. Alternatively, the CFDL platform provides the option of image upload via shell-scripting utilizing a .csv spreadsheet containing labels with associated cloud storage locations. We utilized the latter upload approach for the efficient management of our large datasets. Automated machine learning was then employed, which entails neural architecture search and hyperparameter tuning. Training was performed with maximum allowable cloud graphics processing unit (GPU) hours, and early stopping was enabled, which automatically terminated training when no further model improvement was noted after 581 node-hours. Each node hour represents eight cloud nodes used in parallel, and each node is equivalent to a NVIDIA Tesla V100 GPU. XRAI region based attribution saliency maps were generated from an edge optimized version of the model utilizing the AutoML explainable AI framework^42^.

### Statistical analysis

Statistical analysis was performed with Microsoft Excel and Stata. The model was evaluated at a softmax confidence threshold of 0.5. Area under the precision recall curve (AUPRC) was provided by the platform, and area under the receiver operating characteristic curve (AUROC) was obtained via shell-scripting in order to enable comparison with other reported models. We manually calculated sensitivity, specificity, positive predictive value (PPV), and accuracy (ACC) from confusion matrices provided by the CFDL platform (Tables S3, S4). The former four metrics were calculated with respect to prediction of female sex. For subgroup analysis of model performance on images with foveal pathology, a chi squared test was performed.

## Supplementary Information

Supplementary Information.

## Data Availability

The primary dataset that supports the findings of this study is available, with restrictions, from UK Biobank. The external validation data that support the findings of this study are available on request from the corresponding author PAK. The data are not publicly available due to containing information that could compromise research participant privacy/consent.
