# Supplementary material for: Predicting sex from retinal fundus photographs using automated deep learning
Source: Sci Rep. 2021 May 13;11:10286. doi: 10.1038/s41598-021-89743-x (PMC8119673; doi:10.1038/s41598-021-89743-x)
Supplement: Supplementary file 1 — Supplementary Information. [file 41598_2021_89743_MOESM1_ESM.docx]

### Supplementary Materials

#### Figure S1: Ungradable Images from the Uk Biobank (A) and Moorfields External Validation (B) Datasets


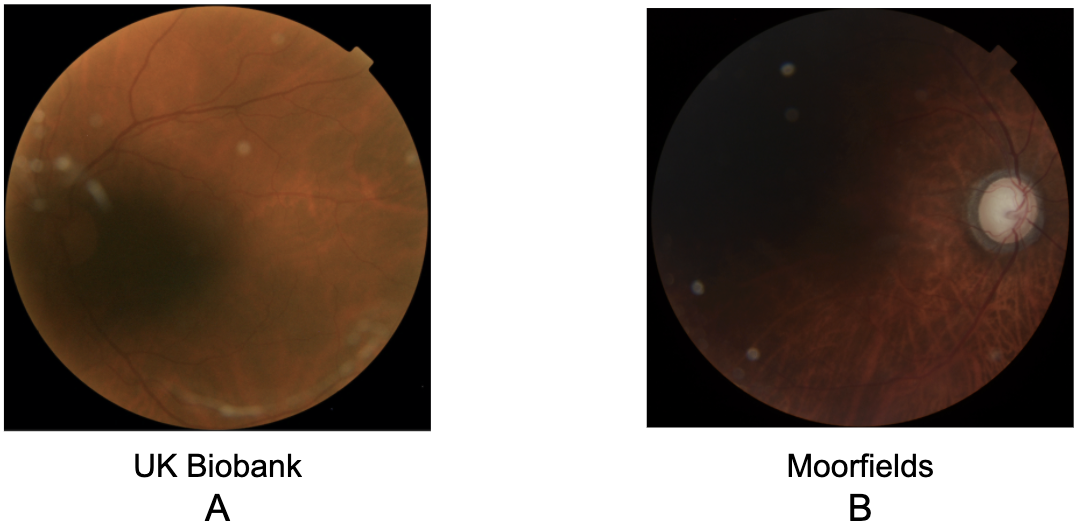


####

####

#### Table S1: Discordant Reported and Genetic Sex in UK Biobank Dataset

|  | **Number of Images** | **Percent of Dataset** | **Total** |
| --- | --- | --- | --- |
| **UK Biobank Train** | 119 | 0.09% | 139,060 |
| **UK Biobank Tune** | 13 | 0.04% | 34,764 |
| **UK Biobank Validation** | 1 | 0.08% | 1,287 |

####

####

####

#### Table S2 Gradability Algorithm Details

| **Model Facts** | | **Model name:** **Retinal Fundus Gradability** | | | |
| --- | --- | --- | --- | --- | --- |
| **Summary**  **This model uses retinal fundus photos as input**  **which were labeled by retinal specialists for gradability,**  **to classify retinal fundus photos as gradable or ungradable.** | | | | | |
| **Mechanism** | | | | | |
|  | **Outcome** | Whether a retinal fundus photo is gradable, defined as:  1. Field of view: Adequate visibility of retinal features (vascular arcades, macula, optic nerve);  2. Image quality: Ability to exclude features to the subtlety level of microaneurysms | | | |
|  | **Output** | Binary classification of gradable or ungradable | | | |
|  | **Target Population** | All adults with retinal photographs | | | |
|  | **Input data source** | EyePACS public fundus dataset (Kaggle) diabetic screening | | | |
|  | **Input data type** | Retinal fundus photographs, expert gradability labels to the same criteria as in Outcome section | | | |
|  | **Training data location** | <https://www.kaggle.com/tanlikesmath/diabetic-retinopathy-resized> | | | |
|  | **Model type** | Neural Network trained with Google Cloud AutoML | | | |
|  | | | | | |
| **Datasets and Performance** | | | | | |
|  | **Prevalence** | **AUPRC** | **PPV**  **@ 0.5 threshold** | **Sensitivity**  **@ 0.5 threshold** | **Cohort** |
| **Training set** | 19.0% | *Not available in Auto ML | *Not available in Auto ML | *Not available in Auto ML | EyePACS Training/Tune:  convenience sampled  from source n=3984 |
| **Validation set** | 20.9% | 0.934 | 0.897 | 0.872 | EyePACS test:  10% random sampled n=398 |

#### Table S3: Confusion Matrix of CFDL Sex Prediction Model Test Set: Rows Correspond to Predictions and Columns to Ground Truth

|  | **Female** | **Male** |
| --- | --- | --- |
| **Female** | 640 | 93 |
| **Male** | 81 | 473 |

#### Table S4: Confusion Matrix of CFDL Sex Prediction Model External Validation Set: Rows Correspond to Predictions and Columns to Ground Truth

|  | **Female** | **Male** |
| --- | --- | --- |
| **Female** | 115 | 32 |
| **Male** | 22 | 83 |

####

### 
